# PP10 Automated Calculation Of Absolute Effects Using The Abseff Program: A Descriptive Study

**DOI:** 10.1017/S0266462325102304

**Published:** 2025-12-29

**Authors:** Carolina Latorraca, Rafael Leite Pacheco, Patricia Parreira, Ana Luiza Cabrera Martimbianco, Verônica Colpani, Isabela Porto de Toledo, Roberta Borges Silva, Camila Monteiro Cruz, Cecília Menezes Farinasso, Rachel Riera

## Abstract

**Introduction:**

One way of communicating the effect of interventions is by converting relative effects, such as relative risk, into absolute effects. Guidelines from the GRADE working group and the Cochrane Handbook for Systematic Reviews of Interventions recommend this conversion for summarizing and concluding evidence syntheses. However, this conversion can lead to errors, and previous research has indicated that inadequacies are frequent in Cochrane reviews that convert hazard ratios to absolute effect measures.

**Methods:**

A descriptive study outlined the development of the abseff program, created using the ado programming language of the Stata software and R. This program was developed in the context of two projects conducted by the Center of Health Technology Assessment, Hospital Sírio-Libanês, São Paulo-SP, Brazil in collaboration with the Ministry of Health, the National Supplementary Health Agency, and the National Council of Justice through the Support Program for Institutional Development of the Unified Health System (PROADI-SUS). Both projects involve drawing up evidence summaries, and the abseff program aims to convert relative effect into corresponding absolute effect measures.

**Results:**

Abseff converts data on relative effects (relative risks, odds ratios, hazard ratios) into absolute effects, including 95 percent confidence intervals (CI). Researchers input a relative effect and its confidence interval with a baseline risk for the calculation. For example, the command abseff rr 0.83 0.63 1.03 50 130 converts a relative risk of 0.83 (95% CI: 0.63, 1.03) with a baseline risk of 50/130 (38%). The result is presented in absolute anticipated effects and absolute risk difference, and calculations account for bases of 100 and 1,000. The program is freely available via Stata and R. For more details access https://rlpacheco.github.io/abseff/; https://github.com/rlpacheco/abseff_R.

**Conclusions:**

Effective communication of evidence can be challenging, and translating scientific knowledge can be facilitated by presenting absolute effect measures to the decision maker. The abseff program standardizes these conversions, minimizing errors and ensuring reproducibility in analyses. Originally created as part of two projects funded by PROADI-SUS, abseff is now available for use by all health technology assessment researchers.

